# Rat-bite Fever, Canada

**DOI:** 10.3201/eid1208.060044

**Published:** 2006-08

**Authors:** Michael E. Schachter, Lindsay Wilcox, Neil Rau, Deborah Yamamura, Shirley Brown, Christine H. Lee

**Affiliations:** * McMaster University, Hamilton, Ontario, Canada;; † Halton Healthcare Services, Oakville-Trafalgar Memorial Site, Oakville, Ontario, Canada;; ‡ MDS Diagnostic Services, Toronto, Ontario, Canada;; § Toronto Public Health Laboratories, Toronto, Ontario, Canada

**Keywords:** Rat-bite fever, *Streptobacillus moniliformis*, *Spirillum minus*, letter

To the Editor: Rat-bite fever was once considered an infection exclusive to children living in poverty; however, dense urban housing and changing pet-keeping practices may be altering this profile (*1**,**2*). To date, ≈200 cases of rat-bite fever have been reported in the United States (*3*), and a recent study reported a 2-fold increased incidence in California during the 1990s (*1*). We report on 2 cases that occurred in Ontario, Canada, in the early 2000s.

The first case occurred in a previously healthy 29-year-old man who was bitten on the finger by a pet rat. The wound healed spontaneously. After 24 hours, fever and emesis developed; 4 days later, diffuse maculopapular rash and migratory arthritis of the knees, ankles, and finger joints ensued. Physical examination showed a maculopapular rash over the lower extremities, an effusion of the left knee, and a warm, erythematous left ankle.

Laboratory investigations showed hemoglobin level of 134 g/L, leukocyte count of 16.0×10^9^/L, and neutrophil count of 13.8×10^9^/L. Aspiration of the knee produced 70 cm^3^ of cloudy fluid; synovial fluid analysis showed 666×10^6^/L leukocytes with a predominance of neutrophils.

Ceftriaxone, 2 g once a day, was given intravenously for 7 days. Although symptoms improved within 24 hours, the effusion recurred within 48 hours of discontinuing the initial course of ceftriaxone. The knee was surgically drained, and ceftriaxone was continued for 5 weeks. Systemic symptoms and the effusion resolved.

The second case occurred in a previously healthy 9-year-old girl who had mucosal contact with a pet rat. She sought treatment after 7 days of generalized maculopapular and pustular rash and 10 days of fever and headache. She had an associated asymmetric, migratory arthritis.

Physical examination showed superficial scratches from the rat; temperature of 39.6°C; heart rate of 102 bpm; swelling, erythema, and decreased range of motion in several joints; and pustular lesions on the soles of the feet.

The patient’s leukocyte count was 8.3×10^9^/L. Synovial fluid from the knee showed 45.5×10^6^/L leukocytes with 89% neutrophils; the culture showed no growth. Gram stains of blood and pustule swabs showed large, pleomorphic, gram-negative bacilli with long filaments and irregular swellings. Growth occurred on the blood culture after 28 hours of aerobic incubation at 35°C in 10% horse serum. Characteristic puff-ball colonies of Streptobacillus moniliformis were seen in supplemented thioglycolate broth.

Identification of the organism was confirmed by using the Sherlock (MIDI Inc., Newark, DE, USA) system. The major cellular fatty acid components of the isolate matched an S. moniliformis reference strain. The patient received penicillin and gentamicin intravenously for 6 days and was discharged home with a 10-day regimen of amoxicillin. One year later, she remained asymptomatic.

Rat-bite fever commonly results from infection with the zoonotic pathogens S. moniliformis and Spirillum minus. S. moniliformis is more common in Western countries, and S. minus predominates in Asia (*3*). S. moniliformis colonizes the nasopharynx of healthy rats (*4*) and is transmitted by the bite or scratch of rats, squirrels, mice, guinea pigs, and, rarely, cats and other rodent predators. Occasionally, it is transmitted by ingestion of contaminated milk or water (*5**,**6*). The site of inoculation with S. moniliformis usually heals before systemic symptoms develop. After the incubation period of 1 to 22 days, patients experience fever, chills, myalgia, headache, and rash. The rash consists of macules, vesicles, and pustules on the extremities; soles and palms are frequently involved. Joint symptoms range from polyarthralgia to migratory polyarthritis with purulent effusions. A nonsuppurative migratory polyarthritis occurs in ≈50% of patients (*5**,**7*). In rare cases, rash and arthritis may be absent (*8*).

When S. minus (a spirochete) is introduced by rat bite, the bite wound initially heals but then ulcerates, followed by regional lymphadenopathy and a distinctive rash of red and purple plaques. Arthritic symptoms are rare (*9*).

Complications of rat-bite fever include destructive joint disease, pericarditis, endocarditis, abscesses, pneumonia, parotitis, pancreatitis, and, rarely, meningitis and amnionitis. Development of endocarditis results in a mortality rate of up to 50% (*5*).

S. moniliformis can be isolated and cultured from synovial fluid, blood, and abscesses. By contrast, S. minus has not been recovered on artificial media but can be seen by using dark-field microscopy with Giemsa or Wright stains.

Laboratory personnel must be notified when rat-bite fever is suspected because S. moniliformis does not grow in a routine sheep blood or MacConkey agar; it requires rat or horse serum, defibrinated blood, or ascitic fluid to sustain growth. Growth of S. moniliformis is inhibited by sodium polyanetholesulfonate, a substance that is added to blood culture bottles to inhibit the antimicrobial action of blood (*4**,**8*).

Optimal treatment for rat-bite fever is penicillin G given intravenously for 7 to 10 days, followed by penicillin V taken orally for 7 days. Alternatively, tetracycline may be used (*5**,**7**,**9*).

Although rat-bite fever is uncommon, it is increasingly seen as a result of changing patterns of urban living and pet-keeping practices. If unrecognized, this infection can have debilitating sequelae and can be life-threatening.
